# The Role of Biofilms Developed under Different Anthropogenic Pressure on Recruitment of Macro-Invertebrates

**DOI:** 10.3390/ijms21062030

**Published:** 2020-03-16

**Authors:** Eva Cacabelos, Patrício Ramalhosa, João Canning-Clode, Jesús S. Troncoso, Celia Olabarria, Cristina Delgado, Sergey Dobretsov, Ignacio Gestoso

**Affiliations:** 1 MARE—Marine and Environmental Sciences Centre, Agência Regional para o Desenvolvimento da Investigação Tecnologia e Inovação (ARDITI), Edifício Madeira Tecnopolo, Piso 0, Caminho da Penteada, 9020-105 Funchal, Madeira, Portugal; 2 OMM—Oceanic Observatory of Madeira, Agência Regional para o Desenvolvimento da Investigação Tecnologia e Inovação, Edifício Madeira Tecnopolo, Piso 0, Caminho da Penteada, 9020-105 Funchal, Madeira, Portugal; 3 Smithsonian Environmental Research Center, 647 Contees Wharf Road, Edgewater, MD 21037, USA; 4 Departamento de Ecoloxía e Bioloxía Animal, Universidade de Vigo, Campus As Lagoas-Marcosende, E-36310 Vigo, Galicia, Spain; 5 Centro de Investigación Marina, CIM Universidade de Vigo, Illa de Toralla, E-36331 Vigo, Galicia, Spain; 6 Department of Marine Science and Fisheries, College of Agricultural and Marine Sciences, Sultan Qaboos University, Muscat 123, Oman; 7 Center of Excellence in Marine Biotechnology, Sultan Qaboos University, Muscat 123, Oman

**Keywords:** diatoms, bacteria, larval recruitment, biofouling, non-indigenous species

## Abstract

Microbial biofilms can be key mediators for settlement of macrofoulers. The present study examines the coupled effects of microbial biofilms and local environmental conditions on the composition, structure and functioning of macrofouling assemblages. Settlement of invertebrates over a gradient of human-impacted sites was investigated on local biofilms and on biofilms developed in marine protected areas (MPAs). Special attention was given to the presence of non-indigenous species (NIS), a global problem that can cause important impacts on local assemblages. In general, the formation of macrofouling assemblages was influenced by the identity of the biofilm. However, these relationships varied across levels of anthropogenic pressure, possibly influenced by environmental conditions and the propagule pressure locally available. While the NIS *Watersipora subatra* seemed to be inhibited by the biofilm developed in the MPA, *Diplosoma* cf. *listerianum* seemed to be attracted by biofilm developed in the MPA only under mid anthropogenic pressure. The obtained information is critical for marine environmental management, urgently needed for the establishment of prevention and control mechanisms to minimize the settlement of NIS and mitigate their threats.

## 1. Introduction

Biofouling is the undesirable attachment and growth of marine organisms on submerged artificial structures that leads to economic, ecological and safety-related negative effects [1,2,3]. For example, hull fouling generates drag resistance of ships moving through water and increases fuel consumption and emissions of greenhouse gases [2]. In aquaculture infrastructures, biofouling restricts water exchange, increases disease risk and causes deformation of cages and structures [3]. Biofouling of ships, as hull fouling or as solid ballast (i.e., with sand, rocks, soil) also facilitates the arrival of non-indigenous species (NIS) into new regions, which poses an enormous risk for the receiving ecosystem in terms of economic and ecological impacts [4,5]. With the increase of global trade, ship traffic has increased dramatically in the last century and, consequently, the risk of new introductions of NIS has increased [5]. As sites that receive maritime trade goods, ports are at high risk of NIS introductions [6], and evidence suggests that macrofouling assemblages on these artificial structures support more NIS compared to natural habitats [7,8]). The degree of trade that a port receives is indicative of the frequency of exposure that the port and surrounding region has to the NIS [9]. Monitoring the presence of NIS and their impacts and looking for potential preventive measures that minimize their settlement are therefore critical for marine environmental management.

In aquatic environments, biofouling on hard substrates starts as a conditioning film, which leads to biofilms and, subsequently, to complex mature macrofouling communities [10,11,12]. Microorganisms rapidly colonize a clean surface and create highly complex and dynamic three-dimensional structures, known as biofilm or microfouling. In the marine environment, the process of biofilm formation is a sequence of phases with several organisms involved (i.e., bacteria, fungi, diatoms, protozoans, larvae and algal spores). During the initial phases, organized assemblages of mixed microorganisms are mainly composed of bacteria and diatoms, typically surrounded by a matrix of extrapolymeric substances [12,13,14], which can positively and/or negatively interact with each other [13]. Bacteria have been found to be the most important microbes on marine surfaces and, by being early colonizers, these cells may determine the structure and function of the mature biofilm [14,15], reached when the surface bioaccumulation attains the saturation value (sensu [12]). The structure and composition of biofilms is directly influenced by biological, environmental, physical and chemical conditions, such as nutrients, temperature or water chemistry [16,17]. Diatoms, particularly, the pennate diatoms, are one of the first colonizers of the biofouling communities in different marine ecosystems [18]. Moreover, cyanobacteria and diatoms are the earliest photoautotrophs to input energy in the biofilms [19].

Microbial films have long been considered to be key mediators for the settlement and the subsequent colonization by macro-organisms in many studies (e.g., [16,20,21,22,23,24]). Biofilms can act as cues for larval and spore settlement of algae, barnacles, polychaetes, ascidians and mussels, inducing larval settlement and accelerating biofouling [25,26]. However, while some studies conclude that biofilms, for instance, enhance the adhesion strength of the ascidian *Phallusia nigra*, the serpulid *Pomatoceros lamarkii*, the barnacle *Semibalanus balanoides* or several mussels [16], other studies showed no effect or inhibitory effects on the settlement of the barnacle *Balanus trigonus* [16,27] or larvae of many other species [21]. Diatom films can also influence larval settlement for the tube-building polychaete *Spirorbis* (see [24]). Extreme cases include the one described for cyprids of *Amphibalanus (Balanus) amphitrite*, which avoid young biofilms but are induced to settle on older biofilms with more diverse microbial composition and abundance [28], or the contradictory results reported by several studies on the settlement of the polychaete tubeworm *Hydroides elegans* (e.g., [29,30]). Although all kinds of responses have been reported, contradictory effects may result from differences in the experimental procedures used to assess the effect of biofilms on macrofouling settlement [31]. So, despite the available information on biofilms, the relationships between biofilms and recruitment of macrofoulers remain unclear [27,32]. This is of special concern in the case of NIS because the presence of particular microbial films can affect their settlement. For instance, the settlement of the non-native bryozoan *Bugula neritina* larvae was affected by the density of bacteria and the identity of diatoms and bacteria in combined biofilms [33].

In this context, the present study examines how the coupled effects of composition and structure of microbial biofilms and the local environmental conditions of ports where they develop can affect composition, structure and functioning of macrofouling assemblages. Our hypotheses are that a) the settlement of native and NIS macrofouling species will be modulated by the presence of particular microbial biofilms; and b) the local environmental conditions will influence both micro and macrofoulers. For that, we investigated how biofilms developed in pristine and impacted habitats affected the settlement of invertebrate larvae over a gradient of human-impacted sites. Additionally, both biodiversity and functioning of macrofouling assemblages were investigated. This information is urgently needed for future optimization of standardized surveys and the establishment of prevention and control mechanisms to mitigate threats posed by NIS.

## 2. Results

### 2.1. Biofilm Characterization

#### 2.1.1. Weight of Biofilm

**Figure 1:**
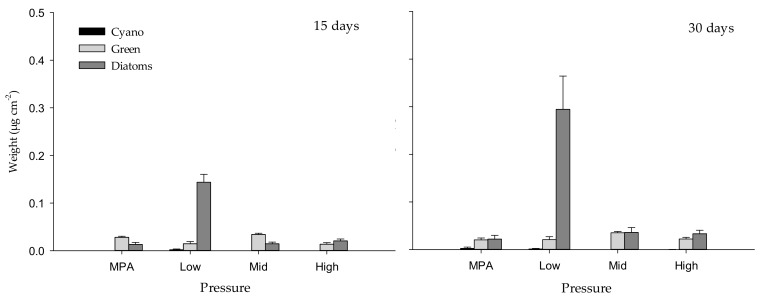
Weight (µg cm^–2^) of cyanobacteria (Cyano), chlorophyll *a* (Green) and diatoms measured with BenthoTorch) among “Pressure” levels after 15 and 30 days of deployment.

**Figure 2:**
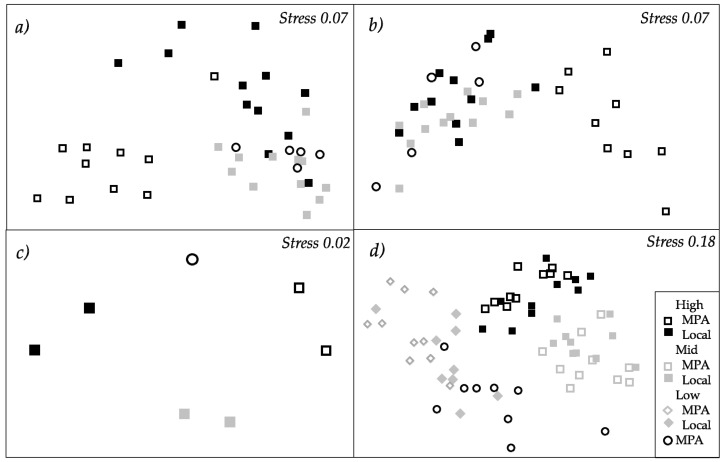
Non-metric multidimensional scaling (nMDS) representing the structure of biofilm assemblages (cyanobacteria, chlorophyll *a* and diatoms measured with BenthoTorch) among “Pressure” levels after (**a**) 15 and (**b**) 30 days of deployment; (**c**) the structure of bacterial assemblages according to different “Pressure” levels after 30 days of deployment; (**d**) the structure of macrofouling assemblages after 15 weeks of deployment. All nMDS are based on Bray–Curtis similarities. Data were fourth-root transformed in (c) and square-root transformed in (d) (for further details, see Table 1, Table 2 and Table 5).

After 15 days, the total assemblage structure of biofilm (including cyanobacteria, green algae and diatoms, some of the main contributors to biofilm formation process) changed significantly with “Pressure”, “Site” and “Panel” (Table 1, Figure 1 and Figure 2a), although the variability among replicates provided a significant contribution to these differences between pressures (Permdisp, *p* < 0.01). While cyanobacteria were absent in almost all pressure levels, green algae reached mean values of approximately 0.014 µg cm^–2^ in sites exposed to high and low anthropogenic pressures, and up to mean values of 0.034 µg cm^–2^ under mid pressure. On the contrary, diatoms reached the maximum values in sites submitted to low anthropogenic pressure, while minimum values were measured under mid and high pressures and in the marine protected area (MPA) (see Figure 1). After 30 days of deployment, values showed a similar trend but greater values, reaching mean values of almost 0.3 µg cm^–2^ in sites submitted to low anthropogenic pressure (see Figure 1). In this case, the effect “Pressure” also showed a significant influence (*p* < 0.01), but the lower dispersion of replicates (Permdisp, *p* > 0.05) did not provide a significant contribution to differences between pressures (Table 1, Figure 1 and Figure 2b).

#### 2.1.2. Bacteria

Biofilm sampled after 30 days of panel deployment revealed that the structure of the bacterial assemblages at the genus level varied significantly depending on anthropogenic “Pressure” (Table 2, Figure 2c), although non-significance was later detected on pair-wise comparisons (Supplementary Materials Table S1). Percentage of similarity increased from 67.97 within low anthropogenic pressure to 70.05 for sites exposed to high pressure and up to 74.44 for mid pressure. On the other hand, dissimilarity was greater between bacterial assemblages established at high vs. low (46.81% dissimilarity) than for the remaining pairs (35.50%–39.40% dissimilarity) (Table 3). Genera contributing most to dissimilarities between high and low pressures were *Francisella* and *Pseudofulvibacter*, being both more abundant under high pressure. *Thiothrix* showed an opposite trend, with greater abundances under low compared to high and mid pressures and even on MPA. *Francisella* also contributed to the dissimilarity between high vs. mid and vs. MPA, as well as *Halocynthiibacter*, which was abundant under high pressure and absent under mid pressure and on MPA. *Alcanivorax* and *Pseudofulvibacter* showed greater abundance under mid vs. low pressure, while *Truepera* showed an opposite tendency, also contributing to the dissimilarity between low vs. MPA, where it was absent. The genus *Proteobacteria* bacterium JGI 0000113-L05 and bacterium WHC4–2, contributed to dissimilarity between high vs. MPA, the later also showing larger abundance under high compared to mid pressure. The genera *Verticia* and *Marivita* showed large abundance under mid pressure and were absent at MPA, while unidentified marine metagenome and uncultured bacterium and prokaryote showed larger abundances at MPA (detailed in Table 3). Taxonomic richness of bacteria varied significantly with “Pressure”, showing higher values at sites exposed to mid pressure (Mean values: 354_Mid_ > 283.5_Low_ = 257_MPA_ = 236_High_).

#### 2.1.3. Diatoms

Due to insufficient material collected in Moaña (mid pressure site), only six sites were used for diatom analysis. A total of 113 diatom taxa were identified; 25 taxa registering abundance over 3% in at least one sample. The structure of diatom assemblages varied significantly across levels of anthropogenic “Pressure” (Table 2), although non-significance was later detected on pair-wise comparisons (Supplementary Materials Table S1). SIMPER analyses (detailed in Table 4) revealed an average dissimilarity between assemblages greater than 57% across all “Pressure” levels. *Grammatophora oceanica* showed greater cover under low anthropogenic pressure than under high or mid pressures, while *Bacillaria socialis* showed the inverse trend. As for *B. socialis, Licmophora flabellata* contributed to the dissimilarity between low vs. mid anthropogenic pressures, being scarcer under low pressure. *Licmophora flabellata* and *Nitzschia* aff. *distans* were the diatoms contributing the most to the dissimilarity between mid and high pressure. While the first was abundant under mid pressure, the latter only appeared in the highly impacted sites. Taxa such as *Achnanthes* aff. *delicatissima* or *Parlibellus* aff. *coxieae* showed great cover in MPA compared to high pressure, where both species were absent.

### 2.2. Structure of Macrofouling Assemblages

A total of 75 taxa were identified across all study sites. Eight were classified as non-indigenous species (NIS), 14 as native to the area, 6 as cryptogenic and 47 as unknown. While the native category (including native species, cryptogenic and taxa with unknown biogeographical status) showed a total mean cover of 68.5%, NIS reached a total mean cover of 5.4%.

**Figure 3:**
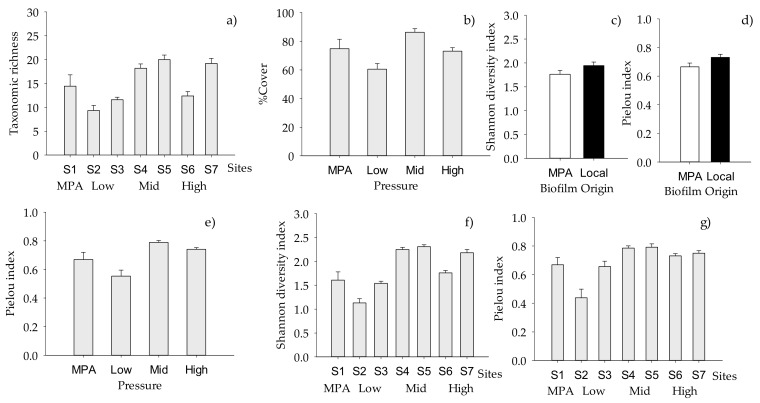
Mean (+SE, per panel) total number of macrofouling taxa across “Sites”; (**a**) (*n* = 10); (**b**) total percentage cover across “Pressure” levels (*n* = 20, *n* = 10 for MPA); values of (**c**) Shannon and (**d**) Pielou indices showing differences between “Origin of biofilm” (*n* = 30 for local, *n* = 40 for MPA) and among “Pressure levels” for (**e**) Pielou index values (*n* = 20, *n* = 10 for MPA); values of (**f**) Shannon and (**g**) Pielou indices showing differences between “Sites” (*n* = 10) (for further details, see Table 5, where significance of factors and interactions are indicated).

Total taxonomic richness of macrofouling did not significantly vary across the “Pressure” or the “Origin of biofilm” (Table 5), but the random factor “Site” had a significant effect, indicating small spatial scale variability in the number of taxa (Figure 3a). The total cover of macrofouling was strongly determined by the anthropogenic “Pressure” (*p* < 0.001), with the maximum and the lowest values of cover at mid and low pressure, respectively (Table 5, Figure 3b). Both Shannon diversity and Pielou evenness indices varied significantly with “Origin of biofilm”. Macrofouling settled on biofilm developed in the MPA presented lower values than those settled on local biofilms (Table 5, Figure 3c,d). In addition, both indices were significantly affected by “Site” (*p* < 0.001 in both cases, Table 5, Figure 3f,g), and Pielou evenness varied significantly across the “Pressure”, showing differences between macrofouling settled in MPA and under Mid pressure (Supplementary Materials Table S2, Figure 3e).

Structure and composition of macrofouling assemblages varied significantly with “Pressure”, but depending on the “Origin of biofilm” (i.e., a significant interaction between factors “Pressure” and “Origin of biofilm”, “Pr × Or”, Table 6a, Figure 2d). A posteriori comparisons showed that macrofouling assemblages settled on local biofilms and on biofilms developed in MPA differed significantly at all “Pressure” levels (Supplementary Materials Table S3). While macrofouling assemblages settled on local biofilms differed between mid and the remaining pressures, differences between assemblages settled on MPA and on biofilms developed in MPA and subjected to low and mid pressures were not detected (Supplementary Materials Table S3).

Moreover, SIMPER analysis (Table 7) revealed average dissimilarities between these assemblages higher than 47% across all “Pressure” levels. While there was more unoccupied space on plates with biofilm developed locally at mid anthropogenic pressure, the percentage of unoccupied space was greater on plates with biofilm developed on the MPA under low and high pressures. Under low pressure, the abundance of cyanobacteria increased twofold in those plates with biofilm developed locally compared to plates with biofilm developed in the MPA. The barnacle *Perforatus perforatus* presented greater cover on biofilm developed locally under low pressure, an opposite trend to that observed under mid and high anthropogenic pressures. Under mid anthropogenic pressure, the tunicates *Ascidia aspersa*, *Botryllus schlosseri*, *Ciona intestinalis* and *Microcosmus* sp. showed greater percentages of cover on biofilm developed locally (Table 7). While *Diplosoma* cf. *listerianum* doubled its abundance on biofilms developed in the MPA compared to those developed locally at mid anthropogenic pressure, the opposite tendency was observed under biofilms developed at high pressure sites. *Spirobranchus triqueter* was more abundant on biofilms developed in the MPA at both mid and high anthropogenic pressure, whereas *Watersipora subatra* had greater cover on biofilms developed locally at high anthropogenic pressure.

**Figure 4:**
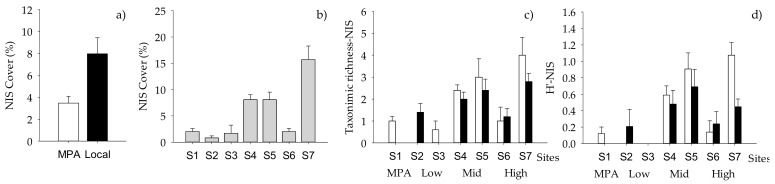
Mean (+SE, per panel) total percentage cover of NIS showing differences between (**a**) “Origin of biofilm” and (**b**) “Sites” (*n* = 10), (**c**) taxonomic richness and (**d**) Shannon index (H’) of NIS showing differences between “Sites” and “Origin of biofilm” (*n* = 5, *n* = 10 for MPA). White bars, MPA biofilm; black bars, local biofilm.

**Figure 5:**
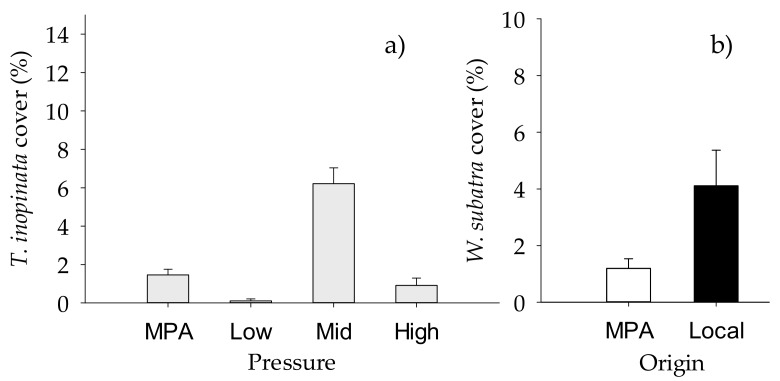
Mean (+SE) percentage cover of NIS (**a**) *Tricellaria inopinata*, showing differences between “Pressure” (*n* = 20, *n* = 10 for MPA), and (**b**) *Watersipora subatra*, showing differences between “Origin of biofilm” (*n* = 30 for local, *n* = 40 for MPA) (*n* = 10). White bars, MPA biofilm; black bars, local biofilm.

When NIS taxa were analyzed separately, the total cover of NIS macrofoulers varied significantly with the “Origin of biofilm” and “Site” (Table 8, Figure 4a,b), while taxonomic richness and diversity index varied significantly with “Origin of biofilm” but depending on “Sites” (Table 8, Supplementary Materials Table S4, Figure 4c,d). The factor “Site” had a significant effect on the percent cover of the most conspicuous NIS, namely *Tricellaria inopinata* and *Watersipora subtatra* (Table 9). In addition, percent cover of *T. inopinata* varied significantly with “Pressure” (Table 9, Supplementary Materials Table S5, Figure 5a), while that of *W. subatra* varied significantly with “Origin of biofilm”, with lower values on biofilm developed in the MPA (Table 9, Figure 5b).

When assemblages of invasive macrofoulers were analyzed separately, there was a significant effect of “Origin of biofilm”, but depending on “Site” (significant interaction “Si(Pr) x Or”, Table 6b). A posteriori comparisons showed that assemblages settled on biofilms developed locally and in the MPA differed significantly at sites located at both high (*p* < 0.01, Náutico site) and low pressure (*p* < 0.05, Museo site) (Supplementary Materials Table S3b).

### 2.3. Functioning of Macrofouling Assemblage

**Figure 6:**
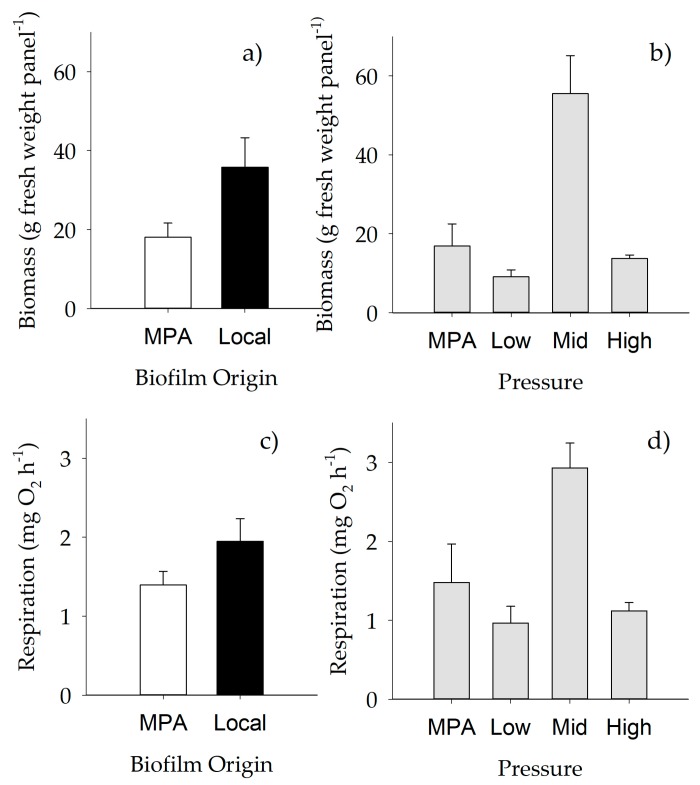
Mean (+SE) biomass (**a**, **b**) and respiration rates (**c**, **d**) across different “Origin of biofilm” (*n* = 30 for local, *n* = 40 for MPA) and “Pressure” levels. (a, c) White bars, MPA biofilm; black bars, local biofilm. For further details, see Table 10.

Biomass of macrofouling assemblages varied significantly with anthropogenic “Pressure” and “Origin of biofilm” (Table 10), with lower values on assemblages settled on biofilms developed in the MPA (Figure 6a), and with strong differences between mid versus high anthropogenic pressure (*p* < 0.01) and mid versus low pressure (*p* < 0.05) (Figure 6b). Similar results were obtained when respiration rate was analyzed, with relevant effects of both anthropogenic pressure and origin of biofilm on respiration per unit of volume (Table 10, Figure 6c,d), and with strong differences between mid versus high and mid versus low anthropogenic pressure.

## 3. Discussion

The present study fully supports our working hypothesis that the settlement of macrofouling species, involving both native and non-indigenous species, was modulated by the coupled effects of composition and structure of microbial biofilms and the local environmental conditions where they develop, demonstrating their relevance in determining the structure and functioning of resulting macrofouling assemblages.

Differences in the environmental context, geographical area or season can mediate changes in the composition and structure of biofilms, which in turn act as key mediators in the subsequent colonization by macrofoulers [16]. Differences in concentrations of diatoms, cyanobacteria and chlorophyll *a* were already detected during the first days of the study, as previously reported in other regions (e.g., [13,14]). Additionally, the distribution of bacterial taxa and structure of bacterial assemblages in one month old biofilms was different in areas submitted to different anthropogenic pressure. For instance, according to [34], more sulphate reducing bacteria appeared in biofilms developed at sites exposed to high anthropogenic pollution, while Alphaproteobacteria, Gammaproteobacteria and Cytophaga/Flavobacteria of Bacteroidetes dominated the community composition at the least impacted sites. We also found Gammaproteobacteria to be extremely abundant at the Toralla site, although they appeared across all pressure levels, and Bacteroidetes were even scarcer under low anthropogenic pressure. Not only variation in pollution but also in biotic and abiotic factors such as production, selective recruitment onto a surface, the activity of consumers, mortality, nutrients and water chemistry may be influencing the composition of these biofilms [35,36,37]. The change in the diatoms assemblages across sites could be also involved in the differences found between biofilms. Not only had the factor “Pressure” affected the structure of these assemblages, as variation at lower scales was also detected (across sites and panels). Changes at these small spatial scales can be determinants for biofilm settlement [18,38]. We used the same type of panels across sites to standardize microtopography and minimize substrate differences, although the substrates are necessarily heterogeneous at the microbial scale [31]. Other abiotic factors than substrate characteristics acting at small scales or possible changes over time could be determining the development of particular biofilms, such as chemical and physical gradients created by turbulence or due to polymer webs, turbulent shear and release of interstitial fluid carried by porous materials. Moreover, biotic factors, such as competition or cooperation among species or grazing pressure, could also play an important role in biofilm development [31,35,39].

Biodiversity and functioning of macrofouling assemblages were influenced by the anthropogenic pressure, with assemblages found under mid anthropogenic pressure being functionally different from the remaining ones. As expected, the settlement of macrofouling species was also affected by the local environmental conditions, since the factor “Site” had a strong effect on most measured responses (both univariate and multivariate) for macrofouling recruitment. Many environmental variables can affect the successional patterns of the colonization on hard substrates, and salinity, temperature or water pollution exert obvious influences on the structure of biological assemblages and, consequently, on their functioning (e.g., [40,41,42]). The selected sites, distributed across the coast of Ria de Vigo, differed moderately in numerous abiotic features, such as temperature, salinity, wave exposure and degree of pollution [43,44,45,46]. Additionally, biological factors such as larval production and mortality [47,48] or predation can vary at this small spatial scale [49,50]. Moreover, macroinvertebrate species are known to affect settlement of other species; i.e., some species may induce settlement of conspecifics while others may inhibit the settlement of heterospecifics (e.g., [51]). Stochastic events could also be involved in obtained macrofouling assemblages, usually influencing community structure in coastal and marine environments (see [41]). All these features can have a major influence on recruitment and successional dynamics of hard bottom communities, not only of biofilm [16,31] but also of macrofouling species (e.g., [52,53,54]).

Recruited macrofouling assemblages, as well as their diversity and evenness indices, were also influenced by the identity of the biofilm, with lower diversity and evenness values detected in assemblages settled on biofilm developed at the MPA. Some studies already found that the settlement of larvae was strongly affected by locally-developed films at a particular site, also suggesting that the density of the microbial film could determine the attractiveness to settling larvae, and that these effects could not be easily decoupled [55]. Moreover, our results are in agreement with many other studies that highlighted the role of specific microbial films as key mediators for the recruitment of some sessile species (e.g., [16,21,22,23,25]).

Wahl et al. [37] already stated about the possibility of interaction between epibiotic biofilms with environmental conditions, highlighting the relevance of the identity of the epibiota, the type of interaction considered, and the prevailing environmental conditions for the formation of host-specific assemblages of bacteria. We found that recruitment of some species (e.g., *Watersipora subatra*) was suppressed by the presence of an MPA biofilm, suggesting that characteristics of the biofilms, as chemical cues or parameters related with community structure or extracellular products, could affect its activity, as already shown by [25] for *Mytilus galloprovincialis*. This supports the capacity of biofilms as key players in determining the colonization process of macrofoulers and, consequently, on the structure and functioning of biological assemblages [16].

Increasing the propagule pressure of NIS, because of marine traffic pressure, is an important factor in controlling the establishment and stability of non-native populations worldwide [56]. We found a lower NIS-cover on panels deployed on the MPA, and although they significantly varied across sites, opposite responses were detected at Náutico versus Davila, both submitted to high anthropogenic pressure. Moreover, instead of the lower diversity and evenness detected in those macrofouling assemblages settled on biofilm developed at MPA, taxonomic richness and diversity of NIS varied significantly with origin of biofilm, but also depending on sampled site. This response could mean a higher susceptibility of these systems to the settlement of NIS, reflecting a lower biotic resistance. Opposite responses were again detected at both sites submitted to high anthropogenic pressure. While greater values of NIS Shannon diversity were detected at Náutico site in those macrofouling assemblages settled on biofilm developed at MPA, in Davila the origin of biofilm had no effect on assemblages. These site effects are possibly due to differences in its exposure and age; while Náutico is an old and very enclosed harbor, resulting in poor water renewal and the corresponding ecological problems, Davila is quite an open environment, not surrounded by structures, having short residence times and presumably lower pollution. Lower NIS recruitment has been observed in open marinas compared with partially enclosed ones [57,58], as enclosed and semi-enclosed marinas have complex circulation patterns, increasing the propagule pressure and the likelihood of settlement of NIS due to higher water residency and limited dispersal of planktonic larvae [57]. Despite this spatial variability, in both harbors the origin of biofilm had an effect when the complete assemblage was analyzed, with taxa such as the native *Spirobranchus triqueter* appearing settled mostly on MPA biofilms and the NIS *Watersipora subatra* on local biofilms.

Harbors as those selected in our study are the kind of widely recognized areas of invasion by NIS [59,60,61]. The number and frequency with which larvae arrive at a destination site over time, or propagule pressure, are important factors in controlling the establishment and stability of non-native populations [56,62]. In general, we found seven species catalogued as NIS in this area. Moreover, six more species were classified as cryptogenic, and many taxa have not been identified to species level, so this list could be easily increased. Although we only detected an effect of pressure on total assemblage when percentage cover of macrofouling was analyzed, total cover of NIS was affected by the origin of biofilm, with the smallest cover of NIS on panels from the MPA. The species most contributing to these differences were *Tricellaria inopinata* and *Watersipora subatra*. In particular, our study detected the capacity of biofilm organisms from pristine habitats in modulating the settlement of particular NIS (e.g., *W. subatra*). However, in view of the observed tendencies across anthropogenic pressure or origin of biofilm (while *T. inopinata* showed greater cover on fouling under mid anthropogenic pressure, *W. subatra* seemed to be inhibited by the biofilm developed in the MPA), we suggest a combined effect of the initial composition of the biofilm and the environmental conditions (influencing, e.g., the propagule pressure locally available or the competitive capacities of the species) in modulating the macrofouling assemblages on marine environments. As previously stated, biofilm can have an important role in larval and spore settlement of organisms (e.g., [16]), but this effect can be dependent on specific traits of the species. For example, *Watersipora* spp. are widely recognized as important fouling organisms found on marine vessels (see [61] and references therein), highly tolerant to copper pollution in antifouling coatings, i.e., metal resistant species, allowing a secondary substrate for the epibiosis of other species to establish (see [63] and references therein). This genus, usually reported in harbors and coastal bays, appears to be expanding its distribution worldwide [61], and seems to be a strong competitor for native biota [64,65,66]. Besides the effect of the origin of biofilm on the settlement of macrofouling species on this highly impacted harbor, percentage cover of bare space was also greater on biofilms developed at the MPA. This could be explained by the lower availability of propagules of native species. For example, the native *Perforatus perforatus* presented a greater cover on MPA biofilm, but only at harbors under mid and high pressure. This is reinforced by the general understanding that highly impacted harbors are not good habitats for native species and are instead populated by highly tolerant introduced species [59]. Observed behaviors can be useful for potential preventive measures that minimize the NIS settlement and are therefore critical for marine environmental management.

The introduction of NIS into the environment represents a great risk due to their potential impacts on the structure and functioning of receiving ecosystems [67]. Our results showed how those assemblages developed on local biofilm panels possess higher capacities of biomass production and respiration rates, being the maximum values in mid impacted harbors/environments. These cascading effects would represent a new scale of impacts beyond the reported consequences on the recruitment of macroinvertebrates. In addition, it evidences the important role that biofilm (or microbiome) could be playing here as an ecological control of the functioning fluxes (i.e., “holobiont”; [68]). Conservation of biofilms developed in pristine habitats, e.g., MPAs, can be therefore of direct relevance for the conservation of coastal ecosystem function, especially considering the unstopped urbanization of marine coastal habitats.

Microbial films of natural or artificial substrates (e.g., ships hulls) have long been considered to be key mediators for the settlement and the subsequent colonization by macro-organisms in many studies [16,20,21,22,23,24], facilitating the spread of NIS. Although biofilm can stimulate settlement in macrofouling species, the inductive cues within a biofilm, as well as the mechanism of induction, are poorly understood. In the present study, a combined effect of the initial composition of biofilm and the environmental context, influencing, for example, the propagule pressure locally available, appeared to modulate the macrofouling assemblages on marine environments, determining both their biodiversity and functioning. To the best of our knowledge this is the first study quantifying total macrofouling assemblages over a gradient of human-impacted sites, from highly pressured harbors to MPA, within the region (but see [69]), and the very first determining the role of the microbial biofilms coupled with the local environmental conditions. Temporal monitoring and studies focused on the mechanism of induction of biofilms would provide vital data and assess possible solutions to policy decisions and management actions. This information is critical for marine environmental management and is urgently needed for the establishment of prevention and control mechanisms to minimize the settlement of NIS and mitigate their threats.

## 4. Materials and Methods

### 4.1. General Approach

**Figure 7:**
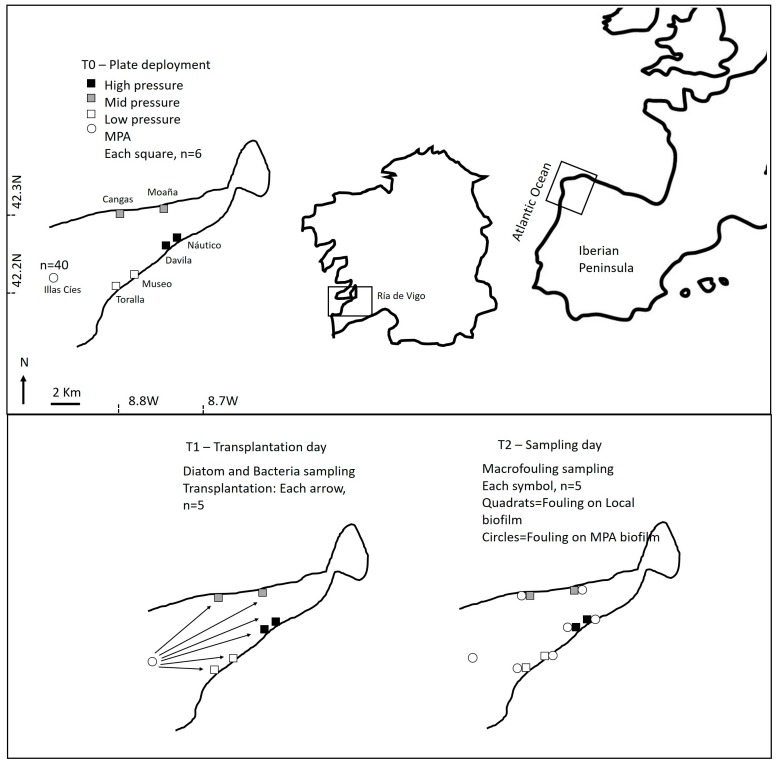
Location of the selected marinas at the Ría de Vigo (NW Iberian Peninsula), and scheme showing the details of experimental procedure and sampling performed during the different phases (T0, T1 and T2).

Several sites distributed across the coast of Ría de Vigo (NW Spain) and exposed to different environmental conditions and anthropogenic pressures (see 43–46), between 2 and 15 km apart, were selected. These sites were grouped into the following four categories: i) a floating pier located within a marine protected area (MPA), i.e., recruiting biofilm assemblage to a stage close to pristine; ii) small harbors without environmental protection, but with almost no maritime traffic, i.e., low pressure; iii) ports affected by medium anthropogenic pressure, including short ferry crossings, aquaculture activities and recreational boats, i.e., mid pressure; and iv) ports affected by high anthropogenic pressures, including fisheries, recreational vessels and commercial traffic, i.e., high pressure. Two suitable experimental sites were selected in each of the selected anthropogenic pressure level categories with the exception of the MPA, where only one marina was available (Figure 7).

Based on the design employed by [70], on 7–8 January 2019, a set of roughened polyvinylchloride (PVC) settlement panels (14 × 14 × 0.3 cm^3^) were individually attached to a brick from buoys or docks between 1 and 2 m depth. Panels were suspended horizontally facing downwards and separated at least 1.5 m within each site. Only the sides of PVC panels facing down were used for measurement and sampling, a standard methodology mimicking floating docks widely used for the study of fouling macroinvertebrates communities [71]. Six panels were suspended at all sites to be colonized by “local” biofilm, whereas 35 additional panels were suspended at the MPA to be colonized by “MPA” biofilm and for posterior experimental transplants (see below).

### 4.2. Biofilm Characterization

#### 4.1.1. Field Sampling

The weight of biofilms (µg cm^–2^) on the side of PVC plates facing down was non-destructively examined 15 and 30 days after the deployment by using a BenthoTorch (bbe-Moldaenke, Germany). This spectrofluorometric tool enables the monitoring and characterization of phytobenthic assemblages directly in the field and is especially indicated for the quantification of biofilm biomass at the early stages of their development [72]. Recorded measures included cyanobacteria, chlorophyll *a* and diatom concentrations (*n* = 3 per panel).

One month after the start of the experiment (4–5 February 2019), which was considered enough time to obtain a mature biofilm (see time scales and stages described in [12] for aquatic biofouling), biofilm assemblages were sampled in order to characterize bacterial and diatom assemblages by using modern genetic and classical taxonomy techniques, respectively. For genetic analysis, a sterile cotton swab was used to carefully swab five settlement panels from each site in order to collect the samples for DNA metabarcoding assemblage (five random panels were selected in the MPA for sampling). Swabs were later transported on ice in a cooler to the laboratory and frozen at –80 °C until further analysis. A replicate panel from each site was sacrificed to be destructively sampled for diatom analysis. All material from the side facing down was scraped with a clean toothbrush and fixed with 4% formaldehyde for further characterization of diatoms using classical taxonomy techniques.

#### 4.1.2. Laboratory Analysis

*Bacteria*. DNA from microbial biofilms was isolated upon arrival to the laboratory using the DNeasy PowerBiofilm Kit (Qiagen) according to the manual. DNA was re-suspended in a final volume of 100 µL. An extraction blank was included in every DNA extraction round and treated as a regular sample to check for cross-contamination. For library preparation, a fragment of the bacterial 16S rRNA region of around 450 bp was amplified using the universal primers 341F R (5’ CCT ACG GGN GGCW GCA G 3’) and 805R (5’ GAC TAC HVG GGT ATC TAA TCC 3’) [73]. The sequences were joined and barcodes were deleted. The sequences <150 bp and with ambiguous base calls were removed. The sequences were de-noised and chimeras were removed. The taxonomy was assigned to ASVs using a pre-trained classifier of the SILVA reference database ([74]; QIIME release 132), using the feature-classifier classify-sklearn approach implemented in QIIME2 [75]. Operational taxonomic units (OTUs) were defined by clustering at 3% divergence and 97% similarity.

*Diatoms*. For analysis of diatom assemblages, the samples were cleaned with 4–6 mL of 65% HNO_3_ and K_2_Cr_2_O_7_ at room temperature for 24–48 h. Afterwards the samples were repeatedly centrifuged (1500 rpm) and rinsed with distilled water to remove oxidation by-products. Finally, permanent slides were mounted using Naphrax^®^ (UK). Two slides (22 × 22 mm^2^) of each sample were examined for diatom content using a light microscope (Olympus BX40, Japan) and a 100× immersion objective (NA 1.25). Diatoms were identified to the lowest taxonomical level possible by using a specialized bibliography [76,77,78,79,80,81,82,83,84,85,86].

### 4.3. Transplantation Experiment

The extra panels deployed at the MPA colonized by pristine biofilms were transplanted to the sites exposed to different anthropogenic pressure (5 units for each site; see Figure 7). For transplantation, panels were collected from the MPA, carefully stowed in containers with seawater to prevent any chafing and transported to each of sites within the following five hours. Five panels were transplanted to each site and ballasted, as previously described. After transplantations, the microfilm assemblages transplanted from the MPA to the respective sites were considered “from MPA”, while the microfilm assemblages from each site were considered “local”.

### 4.4. Macrofouling Characterization

Fifteen weeks after the initial deployment (22–27 April), all panels were retrieved from the field and taken to the laboratory to assess the composition and functioning of macrofouling assemblages. The whole surface of each panel was photographed with an Olympus TG-4 camera (Olympus Corporation, Japan). Panels of each site were maintained separately in 50 L tanks, in an open seawater system with 10 µm filtered UV irradiated seawater at 16–18 °C in an acclimated room (18 °C).

All sessile macroinvertebrates and macroalgae were identified within the following 2 days after collection to the lowest possible taxonomic level with the aid of a stereomicroscope (Olympus SZX9, Olympus Corporation, Japan) and specialized bibliography and then assigned to four categories as follows: native; NIS; cryptogenic (i.e., unknown origin) in accordance with the literature (e.g., [87,88,89,90,91]) and several current databases [92,93,94,95]; or unresolved (unable to identify to species level). Vouchers specimens were collected from the sites and preserved in 95% ethanol or 5% formalin (according to morphotype/taxa) for further identification of dubious records.

For each panel we determined the species richness, total percent cover and bare space by recording the number of species identified from earlier photographs using the image analysis software Coral Point Count (CPCe 4.1; [96]). Consequently, each image was sub-divided into 3 x 3 grids of 9 cells, with 11 random points per cell, resulting in by a matrix of 99 randomly distributed points per picture. This stratified random sampling method ensured that points were sampled in each region of the image [96], and this method has been successfully used in recent similar biofouling sampling analyses (e.g., [63,70]). Fouling organisms were visually identified beneath each cross point up to the highest achievable taxonomic resolution. Organisms present but not falling underneath cross points were recorded as rare and then assigned an arbitrary score of 1%.

We considered the biomass (the wet weight (g), obtained after subtracting the biomass of an empty wet PVC panel from the weight of the colonized one) and respiration as ecosystem functioning surrogates. Respiration rates were estimated by measuring oxygen fluxes during dark periods using 4 L acrylic incubation chambers sealed and submersed in the tanks. Water movement was maintained in the incubation chamber using a submersible pump (300 L·h^–1^; Eheim, type 1001.220, GmbH and Co KG). Variations in oxygen concentration were measured every 30 s using a luminescent dissolved oxygen probe connected to a data-logger (HQ40d Dual-Input Digital Multi-Parameter Meter, Hach, United States). Respiration (measured as mg O_2_ h^–1^) was estimated by regressing oxygen concentration in the chamber over time, with the caution of monitoring chambers without assemblages to correct for potential bacteria and phytoplankton respiration rates. Estimates were also corrected by volume of seawater inside the chamber taking into account the different volumes of assemblages. Water inside the chambers was renewed every new incubation.

### 4.5. Data Analysis

#### 4.5.1. Biofilm

Changes in the weight of biofilm in panels, i.e., µg cm^–2^ of cyanobacteria, green algae and diatoms, were analyzed after 15 and 30 days using permutational multivariate analysis of variance (PERMANOVA) based on untransformed data and Bray–Curtis dissimilarities. The analysis included pressure (fixed, 4 levels: MPA, low, mid, high), site (random, nested in “Pressure”, 2 levels) and panel (random, nested in “Pressure” and “Site”, 5 levels) as factors (*n* = 3). PERMANOVA was also conducted on a square-root transformed percentage of diatoms and on a fourth-root transformed number of OTUs of bacteria observed at the genera level using Bray−Curtis similarities, but in this case including “Pressure” as the only fixed factor. Pair-wise comparisons were done after obtaining a significant factor or a significant interaction of factors. When the number of unique permutations for the pair-wise comparison was less than 30, Monte Carlo *p*-values (P(MC)) were considered [97]. A test for homogeneity of multivariate dispersion (PERMDISP) was performed to complement PERMANOVA [98]. Non-metric multidimensional scaling (nMDS) was conducted for graphical representation of multivariate results [99], and to detect which taxa contributed most to similarity within and dissimilarity among groups, analysis of similarity percentages (SIMPER) was carried out.

#### 4.5.2. Macrofouling Assemblages

For each plate, relevant descriptors of community structure such as species richness, Shannon diversity and Pielou evenness indices were calculated. Since many of the organisms could not be identified to species level, we used the term taxonomic richness rather than species richness. Univariate data such as total percentage cover of macrofoulers and the most conspicuous NIS, taxonomic richness, Shannon index and Pielou evenness, biomass and respiration rates were analyzed using PERMANOVA based on Euclidean distances. The model included “Pressure” and “Site” (as previously described) and “Origin of biofilm” (orthogonal and fixed, 2 levels: MPA and local) as factors. These analyses were also performed considering separately total percentage cover and taxonomic richness of NIS species.

Changes in macrofouling assemblages were examined using PERMANOVA multivariate procedures based on the square root-transformed data and Bray–Curtis dissimilarities following the same previous model. Pair-wise comparisons were performed when significant differences were found. PERMDISP, nMDS and SIMPER analyses were also carried out.

The software PRIMER v6 and PERMANOVA+ for PRIMER (PRIMER-E Ltd., UK) were used for all data analyses [97,100], and SigmaPlot version 12.3 for graph representation.

## Figures and Tables

**Table 1:** Multivariate analysis of variance of total biofilm assemblage across “Pressure” levels (Pr) and “Sites” (Si(Pr)) after 15 and 30 days of “Panel” (Pl(Si(Pr))) deployment. Analyses are based on Bray–Curtis similarity matrices of untransformed data; df, degrees of freedom.

	15 days		30 days	
Source	df	Pseudo-F	df	Pseudo-F
Pr	3	4.551 *	3	6.108 **
Si(Pr)	3	5.888 ***	3	2.831 **
Pl(Si(Pr))	32	2.968 ***	31	2.677 ***
Res	76		76	
Total	114		113	
PERMDISP		P(perm): 0.004		P(perm) ns

* *p* < 0.05, ** *p* < 0.01, *** *p* < 0.001, ns non-significant

**Table 2:** Multivariate analyses of variance of (a) bacteria and (c) diatom assemblages across “Pressure” levels (Pr) after 30 days of panel deployment. Univariate (b) analysis of variance of bacteria assemblage major descriptors. S: taxonomic richness, H’: Shannon diversity index, J: Pielou evenness index. Analyses are based on (a,c) Bray–Curtis similarity matrices of (a) fourth-root and (c) square-root transformed data in multivariate analyses and on (b) Euclidean distances of untransformed data in univariate analyses. Pair-wise comparisons were performed when significant differences were found (see Supplementary Materials Table S1). df, degrees of freedom.

	Bacteria						Diatoms	
	(a) Total Assemblage		(b)	S	H’	J	(c) Total assemblage	
Source	df	Pseudo-F	df	Pseudo-F	Pseudo-F	Pseudo-F	df	Pseudo-F
Pr	3	2.408 **	3	12.642 (*p* = 0.051)	1.712	0.459	3	1.682 *
Res	3		3				2	
Total	6		6				5	

* *p* < 0.05, ** *p* < 0.01.

**Table 3:** SIMPER analysis on bacterial fourth root-transformed data showing the contribution of taxa to the average (Av.) Bray–Curtis similarity (a) and dissimilarity (b) between “Pressure” levels: Marine Protected Area (MPA), Low, Mid and High. Average (dis)similarities are indicated in brackets.

**(a) Low (Av. Similarity: 67.97%)**	**Av.Abund**	**Av.Sim**	**Contrib%**		
Bacteroidia_uncultured	8.64	0.92	1.35		
Bacteroidia_Ulvibacter	6.63	0.69	1.01		
Gammaproteobacteria_Halioglobus	7.11	0.68	1.01		
Gammaproteobacteria_Halioglobus	6.19	0.67	0.98		
Bacteroidia_Aquibacter	6.02	0.67	0.98		
Gammaproteobacteria_Granulosicoccus	6.25	0.64	0.94		
Gammaproteobacteria_Leucothrix	6.51	0.64	0.94		
Bacteroidia_Lewinella	5.99	0.62	0.92		
Alphaproteobacteria_Loktanella	6.42	0.61	0.9		
Bacteroidia_Lutimonas	6.12	0.61	0.9		
Oxyphotobacteria_Minutocellus sp. CCMP1701	5.74	0.61	0.9		
**Mid (Av. Similarity: 74.44%)**	**Av.Abund**	**Av.Sim**	**Contrib%**		
Bacteroidia_Ulvibacter	9.05	0.79	1.07		
Bacteroidia_uncultured	7.58	0.66	0.88		
Gammaproteobacteria_Halioglobus	7.71	0.64	0.86		
Bacteroidia_Aquibacter	6.97	0.61	0.82		
Alphaproteobacteria_Loktanella	6.93	0.6	0.81		
Bacteroidia_Dokdonia	6.84	0.57	0.77		
Gammaproteobacteria_Halioglobus	6.92	0.55	0.74		
Bacteroidia_Winogradskyella	6.41	0.55	0.74		
Gammaproteobacteria_Marinicella	6.24	0.54	0.72		
Bacteroidia_uncultured	5.97	0.52	0.7		
Oxyphotobacteria_Minutocellus sp. CCMP1701	6.37	0.51	0.68		
Alphaproteobacteria_Sulfitobacter	6.22	0.5	0.67		
Bacteroidia_Maribacter	5.57	0.5	0.67		
**High (Av. Similarity: 70.05%)**	**Av.Abund**	**Av.Sim**	**Contrib%**		
Gammaproteobacteria_Francisella	8.52	1	1.43		
Bacteroidia_Pseudofulvibacter	8.2	0.97	1.38		
Bacteroidia_Ulvibacter	8.36	0.95	1.36		
Alphaproteobacteria_Amylibacter	8.87	0.95	1.35		
Alphaproteobacteria_Candidatus Megaira	7.45	0.88	1.25		
Oxyphotobacteria_bacterium WHC4–2	8.15	0.88	1.25		
Bacteroidia_uncultured	7.34	0.84	1.19		
Gammaproteobacteria_Halioglobus	6.4	0.79	1.12		
**(b) High and Low** **(Av. Dissimilarity: 46.81%)**	**High** **Av.Abund**	**Low Av.Abund**	**Av.Diss**	**Diss/SD**	**Contrib%**
Gammaproteobacteria_Francisella	8.52	0	0.5	224.12	1.06
Bacteroidia_Pseudofulvibacter	8.2	1.76	0.38	3.16	0.8
Gammaproteobacteria_Thiothrix	3.63	8.88	0.31	1.33	0.66
Gammaproteobacteria_uncultured	5.15	0	0.3	5.95	0.64
Campylobacteria_Arcobacter	4.94	0	0.29	2.25	0.63
Bacteroidia_Bernardetia	4.62	0	0.27	2.57	0.58
Oxyphotobacteria_Virgulinella fragilis	0	4.66	0.27	4.7	0.58
Gammaproteobacteria_Aliikangiella	4.54	0	0.26	46.84	0.57
Deinococci_Truepera	1	5.42	0.26	3.25	0.56
Alphaproteobacteria_Candidatus Megaira	7.45	3.03	0.26	9.61	0.55
Alphaproteobacteria_Allorhizobium–Neorhizobium–Pararhizobium–Rhizobium	1.04	5.27	0.25	2.41	0.53
Bacteroidia_Mesoflavibacter	4.31	0	0.25	3.7	0.53
Alphaproteobacteria_Roseobacter clade NAC11-7 lineage	4.05	0	0.24	1.86	0.52
Oxyphotobacteria_Podosira stelligera	0	4.06	0.24	5.31	0.51
Bacteroidia_[Polaribacter] huanghezhanensis	4.03	0	0.23	11.67	0.5
Alphaproteobacteria_Jannaschia	5.07	1.16	0.23	2.08	0.49
Oxyphotobacteria_bacterium WHC4-2	8.15	4.2	0.23	3.16	0.49
**High and Mid** **(Av. Dissimilarity: 39.40%)**	**High Av.Abund**	**Mid Av.Abund**	**Av.Diss**	**Diss/SD**	**Contrib%**
Gammaproteobacteria_uncultured	0	6.4	0.33	1.96	0.84
Gammaproteobacteria_Francisella	8.52	2.45	0.31	2.1	0.8
Alphaproteobacteria_Halocynthiibacter	4.08	0	0.21	2.48	0.54
Oxyphotobacteria_bacterium WHC4-2	8.15	4.28	0.2	2.56	0.5
Deltaproteobacteria_Desulfosarcina	0	3.72	0.19	10.2	0.49
Gammaproteobacteria_Verticia	1.45	5.23	0.19	1.61	0.49
Gammaproteobacteria_Marimicrobium	0	3.61	0.19	5.12	0.48
Oxyphotobacteria_Proteobacteria bacterium JGI 0000113-L05	7.11	3.49	0.19	2.79	0.47
Bacteroidia_Kordia	3.44	0	0.18	2.53	0.46
Gammaproteobacteria_Candidatus Thiobios	0	3.45	0.18	12.83	0.45
Alphaproteobacteria_Thalassobius	3.49	0	0.18	2.66	0.45
Bacteroidia_Muriicola	0	3.42	0.18	13.22	0.45
Actinobacteria_Intrasporangiaceae	0	3.39	0.18	20.42	0.45
Gammaproteobacteria_uncultured	1.89	5.2	0.18	1.43	0.44
Thermoanaerobaculia_Subgroup 24	0	3.35	0.17	8.04	0.44
Bacteroidia_Algibacter	0	3.32	0.17	14.55	0.44
Gammaproteobacteria_Perspicuibacter	3.13	0	0.16	2.94	0.42
Bacteroidia_Eudoraea	0	3.16	0.16	22.31	0.42
Bacteroidia_Bernardetia	4.62	1.53	0.16	1.26	0.41
Deltaproteobacteria_Sva0081 sediment group	0	3.14	0.16	8.13	0.41
Verrucomicrobiae_Roseibacillus	1.37	4.46	0.16	1.82	0.41
**Low and Mid** **(Av. Dissimilarity: 36.13%)**	**Low Av.Abund**	**Mid Av.Abund**	**Av.Diss**	**Diss/SD**	**Contrib%**
Gammaproteobacteria_Thiothrix	8.88	3.85	0.25	1.27	0.69
Gammaproteobacteria_uncultured	3.18	6.4	0.23	1.51	0.63
Deinococci_Truepera	5.42	1.17	0.21	3.08	0.58
Gammaproteobacteria_Alcanivorax	0	4.21	0.21	7.79	0.58
Bacteroidia_Pseudofulvibacter	1.76	5.83	0.2	1.94	0.56
Oxyphotobacteria_Podosira stelligera	4.06	0	0.2	5.5	0.55
Gammaproteobacteria_Cycloclasticus	0	4.04	0.2	14.3	0.55
Gracilibacteria_Gracilibacteria	4.46	1.25	0.17	1.22	0.48
Bacteroidia_[Polaribacter] huanghezhanensis	0	3.42	0.17	3.53	0.47
Actinobacteria_Intrasporangiaceae	0	3.39	0.17	46.32	0.46
Gammaproteobacteria_Oleiphilus	0	3.38	0.17	6.85	0.46
Gammaproteobacteria_Verticia	2.2	5.23	0.17	1.33	0.46
Oxyphotobacteria_Phormidesmis ANT.LACV5.1	3.33	0	0.16	8.29	0.46
Campylobacteria_Arcobacter	0	3.23	0.16	3.44	0.44
Gammaproteobacteria_Hydrogenophaga	0	3.2	0.16	7.9	0.44
Bacteroidia_Polaribacter 5	0	3.17	0.16	3.07	0.43
Gammaproteobacteria_Candidatus Tenderia	0	3.14	0.16	4.15	0.43
Alphaproteobacteria_Roseobacter	0	3.09	0.15	20.29	0.42
Gammaproteobacteria_uncultured	0	3.09	0.15	11.82	0.42
Gammaproteobacteria_Coxiella	1.16	4.23	0.15	1.2	0.42
Gammaproteobacteria_Psychromonas	4.02	0.97	0.15	2.53	0.42
**High and MPA** **(Av. Dissimilarity: 35.54%)**	**High Av.Abund**	**MPA Av.Abund**	**Av.Diss**	**Diss/SD**	**Contrib%**
Gammaproteobacteria_Francisella	8.52	0	0.51	309.51	1.45
Gracilibacteria_marine metagenome	0	5.88	0.36	16.02	1
Oxyphotobacteria_bacterium WHC4-2	8.15	2.43	0.34	5.09	0.96
Oxyphotobacteria_Proteobacteria bacterium JGI 0000113-L05	7.11	1.97	0.31	5.64	0.87
Bacteroidia_Bernardetia	4.62	0	0.28	2.09	0.79
Gammaproteobacteria_Aliikangiella	4.54	0	0.27	41.41	0.77
Parcubacteria_uncultured organism	0	4.22	0.25	16.02	0.72
Alphaproteobacteria_Candidatus Megaira	7.45	3.25	0.25	18.95	0.71
Alphaproteobacteria_Halocynthiibacter	4.08	0	0.25	2	0.7
Oxyphotobacteria_environmental clone OCS182	6.61	2.43	0.25	3.3	0.7
Alphaproteobacteria_Roseobacter clade NAC11-7 lineage	4.05	0	0.25	1.52	0.7
Bacteroidia_Muriicola	0	4.04	0.24	16.02	0.69
**Low and MPA** **(Av. Dissimilarity: 35.48%)**	**Low Av.Abund**	**MPA Av.Abund**	**Av.Diss**	**Diss/SD**	**Contrib%**
Deinococci_Truepera	5.42	0	0.31	460.95	0.87
Alphaproteobacteria_Allorhizobium–Neorhizobium–Pararhizobium–Rhizobium	5.27	0	0.3	4.05	0.85
Gammaproteobacteria_uncultured	0	4.72	0.27	578.22	0.76
Gammaproteobacteria_Thiothrix	8.88	4.59	0.25	0.89	0.69
Alphaproteobacteria_Lentibacter	4.15	0	0.24	1.64	0.67
Oxyphotobacteria_Podosira stelligera	4.06	0	0.23	4.5	0.65
Gammaproteobacteria_HTCC5015	1.72	5.49	0.21	1.55	0.61
Oxyphotobacteria_Psammodictyon panduriforme	0	3.7	0.21	578.22	0.6
Gracilibacteria_uncultured bacterium	0	3.65	0.21	578.22	0.59
Gammaproteobacteria_uncultured	0	3.57	0.2	578.22	0.57
Gammaproteobacteria_Perspicuibacter	0	3.55	0.2	578.22	0.57
Alphaproteobacteria_Aliiroseovarius	0	3.52	0.2	578.22	0.57
Oxyphotobacteria_Cylindrotheca closterium	0	3.46	0.2	578.22	0.56
Bacteroidia_Mesoflavibacter	0	3.45	0.2	578.22	0.56
Gracilibacteria_marine metagenome	2.47	5.88	0.19	8.06	0.55
Sericytochromatia_Hyaloperonospora arabidopsidis	0.93	4.33	0.19	2.57	0.55
**Mid and MPA** **(Av. Dissimilarity: 37.24%)**	**Mid Av.Abund**	**MPA Av.Abund**	**Av.Diss**	**Diss/SD**	**Contrib%**
Gammaproteobacteria_uncultured	6.4	0	0.32	1.61	0.87
Gracilibacteria_marine metagenome	0	5.88	0.3	109.31	0.8
Gammaproteobacteria_Verticia	5.23	0	0.27	2.46	0.71
Alphaproteobacteria_Marivita	4.66	0	0.24	5.29	0.64
Parcubacteria_uncultured bacterium	1.15	5.57	0.22	2.66	0.6
Parcubacteria_uncultured prokaryote	1.17	5.58	0.22	2.61	0.6
Alphaproteobacteria_Pseudorhodobacter	4.21	0	0.21	7.5	0.57
Gammaproteobacteria_Alcanivorax	4.21	0	0.21	6.35	0.57
Alphaproteobacteria_Boseongicola	4.15	0	0.21	22.36	0.57
Gammaproteobacteria_Cycloclasticus	4.04	0	0.2	11.71	0.55
Alphaproteobacteria_Albirhodobacter	4	0	0.2	274.32	0.55
Gracilibacteria_Gracilibacteria	1.25	5.24	0.2	2.22	0.54
Gammaproteobacteria_Pseudomonas	3.85	0	0.2	13.15	0.52
Gammaproteobacteria_Enterobacteriaceae	3.8	0	0.19	1.9	0.52
Alphaproteobacteria_Lentibacter	3.78	0	0.19	2.13	0.51
Bacteroidia_Aquibacter	2.28	6.03	0.19	39.9	0.51
Deltaproteobacteria_Desulfosarcina	3.72	0	0.19	9.33	0.51

**Table 4:** SIMPER analysis on diatoms square-root transformed data showing the contribution of taxa to the average (Av.) Bray–Curtis similarity (a) and dissimilarity (b) between “Pressure” levels. Average (dis)similarities are indicated in brackets.

**(a)**	**Low (Av. Similarity: 33.51%)**	**Av.Abund**	**Av.Sim**	**Contrib%**		
	*Cocconeis costata*	3.81	6.41	19.13		
	*Grammatophora oceanica*	3.95	4.68	13.97		
	*Nitzschia aff. inconspicua*	1.6	2.87	8.56		
	*Amphora* sp.1	1.37	2.34	6.99		
	*Grammatophora serpentina*	1.51	2.32	6.92		
	*Nitzschia* aff. *distans*	1.78	2.32	6.92		
	*Gomphonemopsis* sp.	1.19	2.03	6.05		
	*Opephora* sp.1	2.03	1.66	4.94		
	*Ulnaria biceps*	0.97	1.66	4.94		
	**High (Av. Similarity: 59.12%)**	**Av.Abund**	**Av.Sim**	**Contrib%**		
	*Bacillaria socialis*	4.6	7.52	12.72		
	*Nitzschia* aff. *distans*	2.95	4.92	8.32		
	*Navicula* aff. *subagnita*	2.46	3.97	6.72		
	*Amphora* aff. *acutiuscula*	2.32	3.69	6.24		
	*Fragilaria* aff. *investiens*	2.25	3.55	6.01		
	*Nitzschia* aff. *medioconstricta*	2.15	3.55	6		
	*Cocconeis costata*	1.86	3.08	5.2		
	*Amphora* sp.1	1.62	2.71	4.59		
	*Cocconeis* sp.	1.35	2.29	3.87		
	*Nitzschia* aff. *perindistincta*	1.76	2.29	3.87		
	*Astartiella* sp.1	1.15	2.05	3.46		
	*Nitzschia* aff. *laevis*	2.07	2.05	3.46		
	*Plagiotropis* sp.	1.28	2.05	3.46		
	*Gomphonemopsis* sp.	1.26	1.78	3		
**(b)**	**Low and High** **(Av. Dissimilarity: 68.23%)**	**Low Av.Abund**	**High Av.Abund**	**Av.Diss**	**Diss/SD**	**Contrib%**
	*Grammatophora oceanica*	3.95	0	3.91	1.79	5.73
	*Bacillaria socialis*	0.7	4.6	3.82	2.96	5.6
	*Nitzschia* aff. *medioconstricta*	0	2.15	2.07	5.96	3.03
	*Opephora* sp.1	2.03	0	2.04	1.33	2.99
	*Fragilaria* aff. *investiens*	2	2.25	1.94	3.83	2.84
	*Cocconeis costata*	3.81	1.86	1.93	2.01	2.83
	*Nitzschia* aff. *perindistincta*	0	1.76	1.71	2.66	2.51
	*Amphora* aff. *acutiuscula*	0.69	2.32	1.59	3.43	2.33
	*Navicula* aff. *subagnita*	0.99	2.46	1.47	1.2	2.16
	*Achnanthes* aff. *delicatissima*	1.62	0	1.44	0.86	2.1
	*Grammatophora serpentina*	1.51	0	1.43	4.64	2.09
	*Plagiotropis* sp.	0	1.28	1.24	5.08	1.81
	*Navicula* sp.1	1.23	0.98	1.2	2.92	1.76
	*Nitzschia* aff. *distans*	1.78	2.95	1.17	1.41	1.71
	*Amphora* aff. *exilitata*	1.18	0.98	1.12	4.86	1.64
	*Astartiella* sp.1	0	1.15	1.1	9.41	1.62
	*Nitzschia* aff. *laevis*	1.21	2.07	1.1	1.15	1.61
	*Nitzschia* sp.6	0	1.11	1.08	2.66	1.59
	*Halamphora* sp.	1.02	0	1.05	0.86	1.55
	*Ulnaria biceps*	0.97	0	0.94	3.53	1.38
	*Cocconeis* sp.	0.95	1.35	0.94	1.74	1.38
	**Mid and High** **(Av. Dissimilarity: 68.85%)**	**Mid Av.Abund**	**High Av.Abund**	**Av.Diss**	**Diss/SD**	**Contrib%**
	*Licmophora flabellata*	3.23	0	3.14	13.85	4.56
	*Nitzschia* aff. *distans*	0	2.95	2.86	48.77	4.15
	*Navicula* aff. *subagnita*	0	2.46	2.38	16.15	3.45
	*Amphora* aff. *acutiuscula*	0	2.32	2.27	4.47	3.29
	*Nitzschia* aff. *medioconstricta*	0	2.15	2.09	5.74	3.04
	*Nitzschia* aff. *laevis*	0	2.07	2.05	1.46	2.98
	*Seminavis* sp.	1.9	0	1.84	13.85	2.67
	*Nitzschia* aff. *perindistincta*	0	1.76	1.73	2.24	2.51
	*Nitzschia* sp.2	1.51	0	1.47	13.85	2.13
	*Bacillaria socialis*	6.08	4.6	1.41	3.37	2.05
	*Gomphonemopsis exigua*	1.4	0	1.36	13.85	1.97
	*Grammatophora oceanica*	1.4	0	1.36	13.85	1.97
	*Fragilaria* sp.1	1.62	0.29	1.31	2.68	1.9
	*Nitzschia* sp.1	1.71	0.41	1.25	2.68	1.82
	*Plagiotropis* sp.	0	1.28	1.25	4.65	1.81
	*Gomphonemopsis* sp.	0	1.26	1.21	4.46	1.76
	*Astartiella* sp.1	0	1.15	1.12	13.63	1.62
	*Achnanthes* sp.1	1.14	0	1.11	13.85	1.61
	*Lyrella abrupta*	1.14	0	1.11	13.85	1.61
	*Nitzschia* aff. *coarctata*	1.81	0.7	1.11	1.06	1.61
	*Nitzschia* sp.6	0	1.11	1.09	2.24	1.59
	**Low and Mid** **(Av. Dissimilarity: 74.21%)**	**Low Av.Abund**	**Mid Av.Abund**	**Av.Diss**	**Diss/SD**	**Contrib%**
	*Bacillaria socialis*	0.7	6.08	5.7	3.36	7.68
	*Licmophora flabellata*	0.4	3.23	2.94	11.88	3.96
	*Grammatophora oceanica*	3.95	1.4	2.81	1.01	3.79
	*Fragilaria* aff. *investiens*	2	2.06	2.1	6.3	2.83
	*Nitzschia* aff. *coarctata*	0	1.81	1.9	8.58	2.56
	*Nitzschia* aff. *distans*	1.78	0	1.81	2.44	2.44
	*Nitzschia* sp.1	0	1.71	1.8	8.58	2.42
	*Seminavis* sp.	0.29	1.9	1.71	2.76	2.31
	*Fragilaria* sp.1	0	1.62	1.7	8.58	2.29
	*Nitzschia* aff. *inconspicua*	1.6	0	1.69	3.49	2.28
	*Nitzschia* sp.2	0	1.51	1.59	8.58	2.14
	*Achnanthes* aff. *delicatissima*	1.62	0	1.56	0.71	2.1
	*Grammatophora serpentina*	1.51	0	1.56	4.08	2.1
	*Gomphonemopsis exigua*	0	1.4	1.47	8.58	1.98
	*Cocconeis* sp.	0.95	2.21	1.41	0.9	1.9
	*Navicula* sp.1	1.23	0	1.4	0.71	1.89
	*Opephora* sp.1	2.03	0.99	1.37	0.81	1.85
	*Gomphonemopsis* sp.	1.19	0	1.26	2.88	1.7
	*Odontella aurita*	0.4	1.62	1.24	2.79	1.67
	*Nitzschia* aff. *laevis*	1.21	0	1.23	2.69	1.66
	**MPA and High** **(Av. Dissimilarity: 57.14%)**	**MPA Av.Abund**	**High Av.Abund**	**Av.Diss**	**Diss/SD**	**Contrib%**
	*Achnanthes* aff. *delicatissima*	3.51	0	3.06	15.43	5.35
	*Parlibellus* aff. *coxieae*	2.81	0	2.45	15.43	4.28
	*Bacillaria socialis*	1.86	4.6	2.4	3.85	4.2
	*Nitzschia* aff. *laevis*	0	2.07	1.84	1.47	3.22
	*Seminavis* sp.	1.78	0	1.55	15.43	2.71
	*Achnanthes* sp.1	1.59	0	1.38	15.43	2.42
	*Achnanthes* aff. *vistulana*	1.59	0	1.38	15.43	2.42
	*Plagiotropis* sp.	0	1.28	1.12	4.81	1.96
	*Nitzschia* aff. *perindistincta*	0.56	1.76	1.06	1.63	1.85
	*Astartiella* sp.1	0	1.15	1	15.15	1.75
	*Nitzschia* sp.6	0	1.11	0.98	2.28	1.71
	*Caloneis* sp.	1.12	0	0.98	15.43	1.71
	*Licmophora gracilis*	1.12	0	0.98	15.43	1.71
	*Cocconeis* sp.	2.45	1.35	0.96	64.73	1.67
	*Nitzschia* aff. *inconspicua*	2.03	0.98	0.92	3.45	1.6
	*Amphora* aff. *exilitata*	2.03	0.98	0.9	6.03	1.58
	*Nitzschia* aff. *medioconstricta*	1.12	2.15	0.9	3.59	1.57
	*Nitzschia* sp.4	0	0.98	0.86	3.27	1.51
	*Amphora* sp.3	0.97	0	0.85	15.43	1.48
	*Cocconeiopsis* sp.	0.97	0	0.85	15.43	1.48
	*Cyclotella* aff. *atomus*	0.97	0	0.85	15.43	1.48
	*Grammatophora oceanica*	0.97	0	0.85	15.43	1.48
	*Navicula* aff. *cincta*	0.97	0	0.85	15.43	1.48
	**MPA and Low** **(Av. Dissimilarity: 59.22%)**	**MPA Av.Abund**	**Low Av.Abund**	**Av.Diss**	**Diss/SD**	**Contrib%**
	*Grammatophora oceanica*	0.97	3.95	2.89	1.17	4.87
	*Parlibellus* aff. *coxieae*	2.81	0	2.62	9.63	4.42
	*Opephora* sp.1	0	2.03	1.98	1.09	3.34
	*Fragilaria* aff. *investiens*	3.13	2	1.94	1.16	3.28
	*Achnanthes* aff. *delicatissima*	3.51	1.62	1.87	0.81	3.17
	*Cocconeis costata*	1.86	3.81	1.87	1.72	3.15
	*Navicula* aff. *subagnita*	2.69	0.99	1.66	1.13	2.8
	*Cocconeis* sp.	2.45	0.95	1.46	1.05	2.47
	*Seminavis* sp.	1.78	0.29	1.41	2.7	2.38
	*Grammatophora serpentina*	0	1.51	1.39	3.88	2.34
	*Achnanthes* sp.1	1.59	0.29	1.24	2.45	2.09
	*Fragilaria pinnata*	1.26	0	1.17	9.63	1.98
	*Amphora* aff. *exilitata (pediculus)*	2.03	1.18	1.16	0.94	1.95
	*Navicula* sp.1	1.49	1.23	1.13	5.32	1.91
	*Bacillaria socialis*	1.86	0.7	1.13	1.09	1.91
	*Nitzschia* aff. *laevis*	0	1.21	1.1	2.61	1.86
	*Caloneis* sp.	1.12	0	1.05	9.63	1.77
	*Nitzschia* aff. *medioconstricta*	1.12	0	1.05	9.63	1.77
	*Halamphora* sp.	0	1.02	1.02	0.71	1.73
	*Eunotogramma marinum*	0	1.04	0.95	4.45	1.61
	**MPA and Mid** **(Av. Dissimilarity: 70.47%)**	**MPA Av.Abund**	**Mid Av.Abund**	**Av.Diss**	**Diss/SD**	**Contrib%**
	*Bacillaria socialis*	1.86	6.08	3.97	-	5.64
	*Achnanthes* aff. *delicatissima*	3.51	0	3.31	-	4.69
	*Parlibellus* aff. *coxieae*	2.81	0	2.65	-	3.76
	*Navicula* aff. *subagnita*	2.69	0	2.54	-	3.6
	*Licmophora flabellata*	0.79	3.23	2.3	-	3.26
	*Nitzschia* aff. *distans*	2.1	0	1.98	-	2.81
	*Nitzschia* aff. *inconspicua*	2.03	0	1.91	-	2.71
	*Nitzschia* aff. *coarctata*	0	1.81	1.7	-	2.42
	*Nitzschia* sp.1	0	1.71	1.62	-	2.29
	*Fragilaria* sp.1	0	1.62	1.52	-	2.16
	*Odontella aurita*	0	1.62	1.52	-	2.16
	*Achnanthes* aff. *vistulana*	1.59	0	1.5	-	2.12
	*Nitzschia* sp.2	0	1.51	1.43	-	2.02
	*Amphora* aff. *acutiuscula*	1.49	0	1.4	-	1.99
	*Navicula* sp.1	1.49	0	1.4	-	1.99
	*Gomphonemopsis exigua*	0	1.4	1.32	-	1.87
	*Fragilaria pinnata*	1.26	0	1.18	-	1.68
	*Amphora* aff. *exilitata*	2.03	0.81	1.15	-	1.63
	*Lyrella abrupta*	0	1.14	1.08	-	1.53

**Table 5:** Univariate analyses of variance of macrofouling assemblages across “Pressure” levels (Pr), “Origin of biofilm” (Or) and “Sites” (Si(Pr)) after 15 weeks of panel deployment. N: Percentage cover, the remaining abbreviations as in Table 2. Analyses are based on Euclidean distances of untransformed data of S, H’ and J, and on square root transformed data of N. Pair-wise comparisons were performed when significant differences were found (see Supplementary Materials Table S2).

		S	H’	J		N
Pooled terms		Si(Pr) × Or	Si(Pr) × Or	Si(Pr) × Or		Si(Pr); Si(Pr) × Or
Transf.		None	None	None		Square root
Source	df	Pseudo-F	Pseudo-F	Pseudo-F	df	Pseudo-F
Pr	3	2.892	5.982	6.659 *	3	10.482 ***
Or	1	0.009	4.270 *	7.922 **	1	1.573
Si(Pr)	3	6.237 ***	7.954 ***	5.744 **	2	0.902
Pr × Or ^+^	2	2.457	1.362	0.060		
Pooled	58				61	
Total	67				67	
PERMDISP		P(perm): 0.004	P(perm): 0.001	P(perm): ns.		P(perm):0.17

* *p* < 0.05, ** *p* < 0.01, *** *p* < 0.001, ns non-significant, ^+^ Term has one or more empty cells.

**Table 6:** Multivariate analyses of variance of a) total macrofouling assemblage and b) macrofouling assemblage accounting only for non-indigenous species (NIS), across “Pressure” levels (Pr), “Origin of biofilm” (Or) and “Sites” (Si(Pr)) after 15 weeks of panel deployment. Analyses are based on Bray–Curtis similarity matrices of square root transformed data. Pair-wise comparisons were performed when significant differences were found (see Supplementary Materials Table S3).

(a) Macrofouling assemblage			(b) NIS assemblage		
Source	df	Pseudo-F		df	Pseudo-F
Pr	3	4.115 **	Pr	3	3.567 (*p* = 0.07)
Or	1	3.201 ***	Or	1	1.305
Si(Pr)	3	5.320 ***	Si(Pr)	3	8.593 ***
Pr × Or ^+^	2	2.667 ***	PrxOr ^+^	2	0.843
Pooled	58		Si(Pr) × Or	3	2.753 *
			Res	55	
Total	67		Total	67	
PERMDISP	P(perm): ns.				P(perm): 0.001

* *p* < 0.05, ** *p* < 0.01, *** *p* < 0.001, ns non-significant, ^+^ Term has one or more empty cells.

**Table 7:** SIMPER analysis on macrofouling untransformed data showing the contribution of taxa to the average Bray–Curtis (a) similarity within “Pressure” groups and (b) dissimilarity between “Origin of biofilm” across “Pressure” levels. Average (dis)similarities are indicated in brackets.

**(a)**	**Status**	**MPA** **(Av. Similarity: 47.47%)**	**Av.Abund**	**Av.Sim**	**Sim/SD**	**Contrib%**	
	N	*Perforatus perforatus*	34.79	24.86	1.61	52.36	
		Bare space	25.14	13.72	1.13	28.9	
	U	Cyanobact	4.71	1.54	0.44	3.25	
	N	*Spirobranchus triqueter*	2.13	1.18	1.24	2.48	
	N	*Lithophyllum incrustans*	3.37	0.95	0.49	2.01	
	NIS	*Tricellaria inopinata*	1.46	0.95	1.5	2.01	
		**Low** **(Av. Similarity: 48.22%)**	**Av.Abund**	**Av.Sim**	**Sim/SD**	**Contrib%**	
		Bare space	38.92	29.5	2.06	60.94	
	U	Cyanobacteria	23.82	10.46	0.71	21.61	
	N	*Perforatus perforatus*	13.77	4.05	0.46	8.41	
	N	*Lithophyllum incrustans*	4.36	1.69	0.7	3.51	
		**Mid** **(Av. Similarity: 48.80%)**	**Av.Abund**	**Av.Sim**	**Sim/SD**	**Contrib%**	
	C	*Diplosoma* cf. *listerianum*	19.97	12.58	1.47	25.77	
		Bare space	13.74	7.7	1.32	15.78	
	N	*Ciona intestinalis*	9.3	5.05	1.08	10.34	
	C	*Botryllus schlosseri*	9.5	4.43	0.95	9.07	
	NIS	*Tricellaria inopinata*	6.22	4.12	1.76	8.43	
	N	*Perforatus perforatus*	7.33	3.81	1.15	7.8	
	N	*Spirobranchus triqueter*	3.84	2.38	1.4	4.87	
	N	*Ascidia aspersa*	7.53	2.36	0.47	4.83	
	U	*Microcosmus* sp.	2.93	1.4	1.17	2.88	
	NIS	*Watersipora subatra*	1.62	0.88	1.17	1.81	
		**High** **(Av. Similarity: 54.56%)**	**Av.Abund**	**Av.Sim**	**Sim/SD**	**Contrib%**	
		Bare space	26.72	20.63	2.23	37.32	
	N	*Spirobranchus triqueter*	18.23	13.23	2.19	24.25	
	C	*Diplosoma* cf. *listerianum*	12.53	6.15	0.86	11.28	
	N	*Perforatus perforatus*	10.5	4.78	0.84	8.77	
	U	*Foliculina* sp.	8.28	3.9	0.91	7.15	
	NIS	*Watersipora subatra*	6.11	2.19	0.67	4.02	
**(b)**	**Status**	**Low: MPA and Local** **(Av. Dissimilarity: 53.01%)**	**MPA Av.Abund**	**Local Av.Abund**	**Av.Diss**	**Diss/SD**	**Contrib%**
	U	Cyanobacteria	31.01	15.82	14.71	1.31	27.41
	N	*Perforatus perforatus*	7.98	20.2	10.22	1.02	19.27
		Bare space	43.03	34.34	9.97	1.45	18.81
	C	*Kirchenpaueria halecioides*	4.55	5.95	4.16	0.64	7.85
	N	*Lithophyllum incrustans*	3.64	5.16	2.65	1.1	5
	C	*Diplosoma* cf. *listerianum*	0.1	2.81	1.45	0.4	2.73
	**Status**	**Mid: MPA and Local** **(Av. Dissimilarity: 53.07%)**	**MPA Av.Abund**	**Local Av.Abund**	**Av.Diss**	**Diss/SD**	**Contrib%**
	C	*Diplosoma* cf. *listerianum*	26.1	13.84	8.22	1.5	15.48
		Bare space	12.43	15.05	5.86	1.15	11.04
	N	*Ascidia aspersa*	3.44	11.62	5.6	1.13	10.54
	C	*Botryllus schlosseri*	8.49	10.51	4.97	1.11	9.37
	N	*Ciona intestinalis*	6.88	11.72	4.47	1.5	8.43
	N	*Perforatus perforatus*	8.8	5.86	3.47	1.15	6.54
	NIS	*Tricellaria inopinata*	5.36	7.07	2.13	1.42	4.02
	N	*Spirobranchus triqueter*	5.36	2.32	1.71	1.5	3.22
	U	*Microcosmus* sp.	1.62	4.24	1.64	0.92	3.08
	U	*Foliculina* sp.	3.03	1.52	1.61	0.96	3.03
	C	*Kirchenpaueria halecioides*	1.52	2.73	1.28	0.78	2.42
	U	*Cradoscrupocellaria* sp2.	1.01	2.02	1.09	1.01	2.06
	N	*Celleporella hyalina*	1.92	0	0.96	0.87	1.81
	**Status**	**High: MPA and Local** **(Av. Dissimilarity: 47.59%)**	**MPA Av.Abund**	**Local Av.Abund**	**Av.Diss**	**Diss/SD**	**Contrib%**
		Bare space	30.81	22.63	6.65	1.42	13.98
	C	*Diplosoma* cf. *listerianum*	8.59	16.46	6.63	1.38	13.93
	N	*Spirobranchus triqueter*	23.74	12.73	6.16	1.73	12.94
	N	*Perforatus perforatus*	11.11	9.9	5.59	1.22	11.76
	U	*Foliculina* sp.	4.65	11.92	4.79	1.27	10.06
	NIS	*Watersipora subatra*	2.63	9.6	4.26	1.05	8.95
	U	*Bryozoa* sp.1	1.52	1.62	1.25	0.77	2.62
	N	*Cryptosula pallasiana*	1.72	1.11	1.12	0.67	2.36
	U	*Spirorbis* sp.	1.31	1.82	1.06	1.13	2.23
	N	*Celleporella hyalina*	2.12	0.3	1.06	0.84	2.23

**Table 8:** Univariate analysis of variance of non-indigenous (NIS) component of macrofouling assemblage across “Pressure” levels (Pr), “Origin of biofilms” (Or) and Sites (Si(Pr)) after 15 weeks of panel deployment. Analyses are based on Euclidean distances of untransformed data of S and H’, and on square root transformed data of N-NIS. Pair-wise comparisons were performed when significant differences were found (see Supplementary Materials Table S4). Abbreviations as in Table 2 and Table 5.

		N-NIS			S-NIS	H’-NIS
Pooled terms		Si(Pr) x Or				
Transf		Square-root				
Source	df		Source	df	Pseudo-F	Pseudo-F
Pr	3	1.870	Pr	3	1.645	2.366
Or	1	14.246 ***	Or	1	0.031	0.696
Si(Pr)	3	27.484 ***	Si(Pr)	3	13.552 ***	7.051 ***
Pr × Or ^+^	2	1.718	PrxOr ^+^	2	0.842	0.713
Pooled	58		Si(Pr)xOr	3	3.1524 (*p* = 0.05)	2.530 (*p* = 0.07)
Total	67		Res	55		
			Total	67		
PERMDISP		P(perm): 0.001			P(perm): 0.006	P(perm): 0.002

*** *p* < 0.001, MC = Monte Carlo, ^+^ Term has one or more empty cells.

**Table 9:** Univariate analysis of variance of most conspicuous NIS across “Pressure” levels (Pr), “Origin of biofilm” (Or) and “Sites” (Si(Pr)) after 15 weeks of panel deployment. Analyses are based on Euclidean distances of square root transformed percent covers of *Tricellaria inopinata* and *Watersipora subatra*. Pair-wise comparisons were performed when significant differences were found (see Supplementary Materials Table S5).

Tricellaria inopinata		Watersipora subatra			
Pooled terms:		Si(Pr) x Or; Pr × Or			
Source	df	Pseudo-F	Source	df	Pseudo-F
Pr	3	13.701 *	Pr	3	1.345
Or	1	0.005	Or	1	7.561 *
Si(Pr)	3	4.446 **	Si(Pr)	3	18.375 ***
Pooled	5	2.235	Pr × Or ^+^	2	3.651
Res	55		Si(Pr) x Or	3	1.607
Total	67		Res	55	
			Total	67	
PERMDISP	P(perm): 0.001			P(perm): 0.002	

* *p* < 0.05, ** *p* < 0.01, *** *p* < 0.001, ^+^ Term has one or more empty cells.

**Table 10:** Univariate analysis of variance of biomass and respiration rates across “Pressure” levels (Pr), “Origin of biofilm” (Or) and “Sites” (Si(Pr)) after 15 weeks of panel deployment. Analyses are based on Euclidean distances. Pair-wise comparisons were performed when significant differences were found (see Supplementary Materials Table S6).

		Biomass	Respiration Rate
Pooled terms		Si(Pr); Si(Pr) x Or	Si(Pr); Si(Pr) x Or
Transf.		Fourth root	None
Source	df	Pseudo-F	Pseudo-F
Pr	3	26.520 ***	16.233 ***
Or	1	14.990 ***	4.590 *
Pr × Or ^+^	2	2.434	0.853
Pooled	56		
Total	62		
PERMDISP		P(perm): ns	P(perm): 0.026

* *p* < 0.05, *** *p* < 0.001, ns non-significant, ^+^ Term has one or more empty cells.

## Supplementary Materials

The following are available online at https://www.mdpi.com/1422-0067/21/6/2030/s1. Table S1. Pair-wise comparisons performed when significant differences in the univariate and multivariate analyses of variance of bacteria and diatom assemblages across “Pressure” levels (= Pr) after 30 days of panel deployment were found (see Table 2). S: taxonomic richness. Table S2. Pair-wise comparisons were performed when significant differences in univariate analyses of variance of macrofouling assemblages across “Pressure” levels (= Pr) after 15 weeks of panel deployment were found (see Table 5). J: Pielou evenness; N: Percentage cover. Table S3. Pair-wise comparisons were performed when significant differences in multivariate analyses of variance of a) total macrofouling assemblage and b) macrofouling assemblage accounting only for non-indigenous species, across “Pressure” levels (Pr), “Origin of biofilm” (Or) and “Sites” (Si(Pr)) after 15 weeks of panel deployment were found (see Table 6). Table S4. Pair-wise comparisons were performed when significant differences in univariate analysis of variance of non-indigenous (NIS) component of macrofouling assemblage across “Pressure” levels (Pr), “Origin of biofilms” (Or) and Sites (Si(Pr)) after 15 weeks of panel deployment were found (see Table 8). S: taxonomic richness, H’: Shannon diversity index. Table S5. Pair-wise comparisons performed after significant differences in univariate analysis of variance of Tricellaria inopinata across “Pressure” levels (Pr) after 15 weeks of panel deployment were found (see Table 9). Table S6. Pair-wise comparisons performed when significant differences in the univariate analyses of variance of biomass and respiration rates across “Pressure” levels (Pr) after 15 weeks of panel deployment were found (see Table 10).

## Abbreviations

MPA	Marine protected areas
NIS	Non-indigenous species
Pr	Pressure
Or	Origin of biofilm
Si	Site
Pl	Panel
S	Taxonomic richness
H’	Shannon diversity index
J	Pielou evenness index
N	Percentage cover
PERMDISP	Test for homogeneity of multivariate dispersion
SIMPER	Similarity percentages
nMDS	Non-metric multidimensional scaling
PERMANOVA	Permutational multivariate analysis of variance
MC	Monte Carlo
