# Supplementary material for: The Role of Biofilms Developed under Different Anthropogenic Pressure on Recruitment of Macro-Invertebrates
Source: Int J Mol Sci. 2020 Mar 16;21(6):2030. doi: 10.3390/ijms21062030 (PMC7139543; doi:10.3390/ijms21062030)
Supplement: Supplementary file 1 [file ijms-21-02030-s001.pdf]

Table S1. Pair-wise comparisons performed when significant differences in the univariate and multivariate analyses of variance of bacteria and diatom assemblages across "Pressure" levels (= Pr) after 30 days of panel deployment were found (see Table 2). S: taxonomic richness.

	**Bacteria**		**Diatoms**
	**Total assemblage**	**S**	**Total assemblage**
Groups	t, P(perm)	t, P(perm)	t, P(MC)
High, Low	1.892	1.945	1.450
High, Mid	1.742	4.912*	1.857
High, MPA	1.249	0.505	1.507
Low, Mid	1.458	15.294**	1.153
Low, MPA	1.143	3.400	0.884
Mid, MPA	1.581	56.003*	No test

*$p < 0.05$, **$p < 0.01$, MC = Monte Carlo

Table S2. Pair-wise comparisons were performed when significant differences in univariate analyses of variance of macrofouling assemblages across "Pressure" levels (= Pr) after 15 weeks of panel deployment were found (see Table 5). J: Pielou evenness; N: Percentage cover.

	**J**	**N**
Groups	t, P(perm)	t, P(perm)
Mid, MPA	6.863***	1.907
Mid, High	3.096	8.832***
Mid, Low	3.575	5.540***
MPA, High	2.208	0.916
MPA, Low	0.704	2.213*
High, Low	0.491	2.758**

$p < 0.05$, ** $p < 0.01$, *** $p < 0.001$.

Table S3. Pair-wise comparisons were performed when significant differences in multivariate analyses of variance of a) total macrofouling assemblage and b) macrofouling assemblage accounting only for non-indigenous species, across "Pressure" levels (Pr), "Origin of biofilm" (Or) and "Sites" (Si(Pr)) after 15 weeks of panel deployment were found (see Table 6).

**a) Macrofouling assemblage**			**b) NIS assemblage**	
Term "Pr x Or" for pairs of levels of factor "Pressure"			Term "Si(Pr) x Or" for pairs of levels of factor "Origin"	
	**Groups**	t, P(MC)	Groups	t, P(perm)
**Within level MPA of factor "Origin"**	Mid, MPA	2.173*	S2-Toralla	0.882
	Mid, High	1.784*	S3-Museo	1.961*
	Mid, Low	3.209**	S4-Cangas	0.552
	MPA, High	1.316	S5-Moaña	0.997
	MPA, Low	1.687	S6-Davila	1.685
	High, Low	2.188*	S7-Náutico	2.644**
	Groups	t, P(perm)		
Within level Local of factor "Origin"	Mid, High	1.933*		
	Mid, Low	2.672**		
	High, Low	1.762		
Term "Pr x Or" for pairs of levels of factor "Origin"				
	Groups	t, P(perm)		
Mid	MPA, Local	1.661*		
High	MPA, Local	1.730**		

Low	MPA, Local	1.888*

*$p < 0.05$, **$p < 0.01$, MC = Monte Carlo.

Table S4. Pair-wise comparisons were performed when significant differences in univariate analysis of variance of non-indigenous (NIS) component of macrofouling assemblage across "Pressure" levels (Pr), "Origin of biofilms" (Or) and Sites (Si(Pr)) after 15 weeks of panel deployment were found (see Table 8). S: taxonomic richness, H': Shannon diversity index.

	**S-NIS**	**H'-NIS**
Term "Si(Pr) x Or" for pairs of levels of factor "Origin"		
	t, P(MC)	t, P(MC)
Toralla	0.88192	-
Museo	0.009 **	1
Cangas	0.649	0.542
Moaña	0.528	0.747
Davila	1.0954	0.500
Náutico	1.3131	3.470**

**$p < 0.01$, MC = Monte Carlo

Table S5. Pair-wise comparisons performed after significant differences in univariate analysis of variance of *Tricellaria inopinata* across "Pressure" levels (Pr) after 15 weeks of panel deployment were found (see Table 9).

Tricellaria inopinata	
Term "Pr"	
Groups	t, p(MC)
Mid, MPA	3.004
Mid, High	3.946 (P(MC)=0.06)
Mid, Low	10.97 **
MPA, High	0.476
MPA, Low	7.312 *
High, Low	1.040

*$p < 0.05$, **$p < 0.01$, MC = Monte Carlo

Table S6. Pair-wise comparisons performed when significant differences in the univariate analyses of variance of biomass and respiration rates across "Pressure" levels (Pr) after 15 weeks of panel deployment were found (see Table 10).

	**Biomass**	**Respiration rate**
Groups	t, P(perm)	t, P(perm)
MPA, High	0.963	1.177
MPA, Low	1.820	1.623
MPA, Mid	2.008	1.464
High, Low	1.799	0.531
High, Mid	10.255**	5.860***
Low, Mid	5.531*	5.257***

*$p < 0.05$, **$p < 0.01$, ***$p < 0.001$
